# Trauma consultations in a Swiss tertiary emergency department: Comparison of asylum seekers and the local population—Patient characteristics and patterns of injuries, a retrospective study

**DOI:** 10.1371/journal.pone.0277418

**Published:** 2022-11-14

**Authors:** Anne Jachmann, Rabia Saffuri, Henk Eijer, Adam D. Brown, Evika Karamagioli, Emmanouil Pikoulis, Aristomenis Exadaktylos, Karsten Klingberg, David Srivastava

**Affiliations:** 1 Emergency Department, University Hospital Bern, Bern, Switzerland; 2 Medical School, National and Kapodistrian University of Athens (NKUA), Athens, Greece; 3 Department of Orthopedic Surgery, Spital Emmental, Burgdorf, Switzerland; 4 Department of Psychology, New School of Social Research, New York, NY, United States of America; 5 Department of Psychiatry, New York University School of Medicine, New York, NY, United States of America; Universitat de Valencia, SPAIN

## Abstract

**Background:**

In 2017, accidents and other acts of violence were the fifth most common cause of death in Switzerland. Moreover, there are increasing numbers of refugees and asylum seekers (AS), who often exhibit distinct disease profiles from those of the natives of the host country. If these differences could be clearly identified, this might help to develop and implement strategies to prevent injuries in health care programs for refugees and asylum seekers. The aim of this study was to examine the types and characteristics of physical trauma profiles in patients from the two largest groups of AS in Switzerland–from Eastern Africa (EA) and the Middle East (ME)–who consulted a Swiss Emergency Department (ED) in 2017/2018. Furthermore, the physical trauma profiles of Swiss national (SN) patients were examined in order to explore potential differences.

**Methods:**

Descriptive retrospective study of adult trauma patients consulting the ED of a Swiss University Hospital between 01/2017 and 12/2018. The study included 157 asylum seeking trauma patients from EA and ME were included in the study. These were matched by gender and age to 157 Swiss trauma patients consulting the ED in the study period.

**Results:**

There were significant differences between the groups with respect to type of admission, level of severity, localization and mechanisms of injury. While SN had higher levels of injuries related to road traffic or work, AS had higher levels of injuries related to attempted suicide or to assault.

**Conclusions:**

There were differences between AS and the local population with respect to the characteristics and patterns of injury, so that strategies for preventing injuries and promoting health must be tailored to the target population. Moreover, the observed high rates of outpatient treatment for both groups underline the increasing role of EDs as primary care providers for the population served.

## Background

In the light of numerous crises and conflicts in the Middle East and Africa, the number of migrants is still high, and there have therefore been enormous increases in the number of Asylum Seekers (AS) to Europe, including Switzerland and other places in the world.

With 1.9 asylum seekers per 1000 inhabitants in 2018 (previous year: 2.2), Switzerland remains well above the European average of 1.2 asylum seekers per 1000 inhabitants [1]. In 2018, the largest group of asylum seekers arriving in Switzerland were from Eritrea. Further important countries were Syria, Afghanistan and Turkey [1].

With the increases in the numbers of persons seeking asylum in Europe and Switzerland, the role they are playing in healthcare systems is becoming more important and challenging, especially in preventive healthcare. Studies have shown that the asylum process itself, the uncertainty on the outcome of the application and the restricted living regulations lead to significant effects on the health of asylum seekers [2, 3].

Although traumatic injuries are among the leading causes of morbidity and mortality, especially in the age group of under 65 years [4], the vast majority of studies concentrate on other aspects of health care, such as the mental health of refugees and asylum seekers [5–8]. There have been very few studies on traumatic injuries suffered by asylum seekers and refugees which include comparisons with the host population [9], and most of these studies have focused on the type of injuries that refugees experienced during war in their home countries [10].

According to the “Bundesamt für Statistik” (Swiss Federal Bureau for Statistics) in 2017, “accidents and other acts of violence” were the fifth most frequent cause for death in Switzerland, surpassed only by cancer, cardiovascular disease, dementia and diseases of the respiratory system [4]. Accidents are one of the main causes of premature mortality and a frequent reason for hospitalization. In 2017 alone, 141,084 people were admitted in hospitals as a result of an accident in Switzerland [11]. Since these are basically preventable, they represent a key challenge for public health and prevention.

These statistics do not focus on populations seeking asylum. However, in the view of increasing numbers of AS and possible differences in their disease profiles from those of the host population [2, 12], it is essential for a holistic healthcare program, to explore and understand also injury patterns in AS. Identifying potential differences in injury patterns compared to the population in the host country, might help to develop and implement strategies for the prevention of injuries within programs for the health care of AS.

Studies have shown that emergency departments (EDs) act as central contact points and primary care providers for AS and face increasing numbers of consultations [13, 14]. In AS, traumatic injuries are among the most common ED presentations [15].

Therefore the aim of this study was to examine the types and characteristics of consultations at the Emergency Department of the University Hospital in Bern with respect to physical trauma profiles among the two dominant and most frequent groups of AS in Switzerland–from Eastern Africa (EA) and the Middle East (ME) -. These regions were identified from the Swiss asylum statistics, as three quarters of all people in the asylum process in Switzerland in 2018 originated from countries in these regions [1]. Furthermore, the traumatic injury patterns of Swiss national (SN) patients were examined in order to explore potential differences. Insights into patient characteristics and patterns of injuries within different populations might guide policy makers in establishing population-based public health and safety interventions.

## Methods

We conducted a retrospective, single center study at the ED of the University Hospital in Bern (Inselspital). With a catchment area of 2 million people, the University Hospital in Bern is one of the largest Swiss hospitals. In 2020, over 50,000 patients were treated in the ED. Data were analyzed for a two year period between January 2017 and December 2018.

The study included adult patients, of 18 years and above, who were from the two largest and most frequent groups of AS in Switzerland and who consulted the ED between 2017 and 2018 with traumatic injuries. The country of origin, the resident status and the asylum status are routinely registered in the hospital administration. The classification of the nationality to the geographical region (Middle East (ME) and Eastern Africa (EA)) was made according to the online platform for Geography “Lexas” (Table 1) [16, 17].

**Table 1:** Countries divided into geographical regions.

**Middle East**	Egypt, Bahrain, Gaza Strip, Iraq, Iran, Israel, Yemen, Jordan, Qatar, Kuwait, Lebanon, Oman, Saudi Arabia, Syria, Turkey, United Arab Emirates, West Bank, Cyprus
**East Africa**	Ethiopia, British Indian Ocean Territory, Burundi, Djibouti, Eritrea, French Southern and Antarctic Territories, Kenya, Comoros, Madagascar, Malawi, Mauritius, Mayotte, Mozambique, Réunion, Rwanda, Zambia, Seychelles, Zimbabwe, Somalia, South Sudan, Tanzania, Uganda

In order to create a comparison group, each AS study patient was matched with the next Swiss national patient admitted to the ED because of a traumatic injury after the study patient. In addition, study and comparison patients were matched by gender and age (+/- 5 years). Patients with other nationalities were not included in the study.

For the patients who met the inclusion criteria, the electronical medical records (electronical database: E-Care, Turnhout, Belgium) were reviewed and the following demographical and clinical data were extracted: Patient age, grouped into three categories: 18–30, 31–60, > 60; gender (female, male); type of referral (ambulance, helicopter emergency medical service, outpatient walk-in emergency practice, self-admission, external hospital, general practitioner); length of stay (LOS) in the ED; discharge status (home, hospitalization, relocation to an external hospital) and triage category of the Swiss Triage Scale (STS), which classifies the urgency for treatment in five levels: 1 = acute life threatening problem to 5 = non urgent condition [18].

After reviewing several trauma classifications, including the ICD 10 code, we classified the trauma in various categories. The localization of the trauma was grouped into: head / face / neck, thorax, abdomen and pelvis, lower and upper extremities, and multiple locations. Types of injuries were summarized under fracture, concussion, cuts / bites / open wound, bruises / superficial, sprain / strain / dislocation, others and multiple injuries. The mechanism of injury was categorized into road-traffic injuries, sports, fall, accidents in everyday life / leisure (e.g. injuries due to garden/kitchen work), accidents at work, assault (e.g. interpersonal violence, sexual abuse), attempted suicide and others. According to the classification of Negussie et al. [19] the severity of injury was grouped into three categories based on the medical care needed: minor/ superficial (minor care, e.g. wound cleaning), moderate (skilled treatment e.g. fracture stabilization) and severe (intensive care, e.g. control of major bleeding). The intentionality of the injury was grouped in three categories, as unintentional, intentional and undetermined. Additionally, the influence of substance use related to the injury was recorded (alcohol, substance (other drug than alcohol), mixed substances and no substance). This information is documented in the medical record, if the patient had indicated it or if this was detected in blood or urine tests.

The data were collected using Microsoft Office Excel 2016 for Windows 10 (Version 1805, Microsoft Corporation, Redmond, Washington, United States). The distribution of categorical data is provided in numbers and percentages and continuous variables as medians with interquartile ranges (IQR). Unless otherwise stated, the chi squared test was used to test for significant differences between the study and comparison groups for categorical variables and the Mann–Whitney U test for continuous variables, as these were not normally distributed.

The study was conducted in accordance with the Declaration of Helsinki. Ethical approval was obtained by the responsible ethics committee (Kantonale Ethikkommision für die Forschung, Bern, Switzerland, 2020–01462), which approved the use of health-related personal data also in the absence of consent. Patients who refused or withdrew their consent to data use were excluded from the analyses.

## Results

In total, 157 AS met the inclusion criteria and were included in the analysis. The comparison group consisted of 157 SN, who were matched for age (+/-5 years) and gender. The demographic characteristics of the groups are summarized in Table 2. The AS patients were from two geographical regions, with 47.8% from the Middle East and 52.2% from Eastern Africa. About 79.6% were males in the AS group. Most were 30 years of age or younger (66.6%).

**Table 2:** Study population demographic characteristics (ME = Middle East; EA = Eastern African; CH = Switzerland).

		Number	Percent
**Age**	18–30	209	66.6
	31–60	103	32.8
	> 60	2	0.6
**Nationality**	ME	75	23.9
	EA	82	26.1
	CH	157	50.0
**Gender**	Female	64	20.4
	Male	250	79.6
	Total	314	100.0

The triage level did not differ (p = 0.225) between the two groups, with a median of 3 (AS–IQR: 3–3; SN–IQR: 3–3). Moreover, there was no significant difference between the two groups in the length of stay (LOS) in the ED (p = 0.626); the median LOS of the SN comparison group was 3:19 h (IQR: 2:10–4:57 h) and of the AS group 3:09 h (IQR: 1:52–5:19 h).

There were significant differences between the groups in the distribution of the level of severity (p = 0.006), with the majority of the SN group having a moderate level of severity (N = 100 (63.7%)) and fewer cases of minor (N = 48 (30.6%)) and severe traumatic injury (N = 9 (5.7%)), whereas the AS group had more cases of minor injuries (N = 70 (44.6%)), a few more cases of severe traumatic injuries (N = 15 (9.6%)) and fewer cases of moderate severity (N = 72 (45.9%)).

Furthermore, the degree of intention had a significantly different distribution within the groups (p < 0.001), with higher levels of unintentional trauma within the Swiss patients group (87.3%) versus the AS patients (61.1%) and higher levels of intentional trauma within the AS group (33.1%) versus the SN group (12.1%), with fewer cases of undetermined intention (AS 5.7%, SN 0.6%).

**Fig 1:**
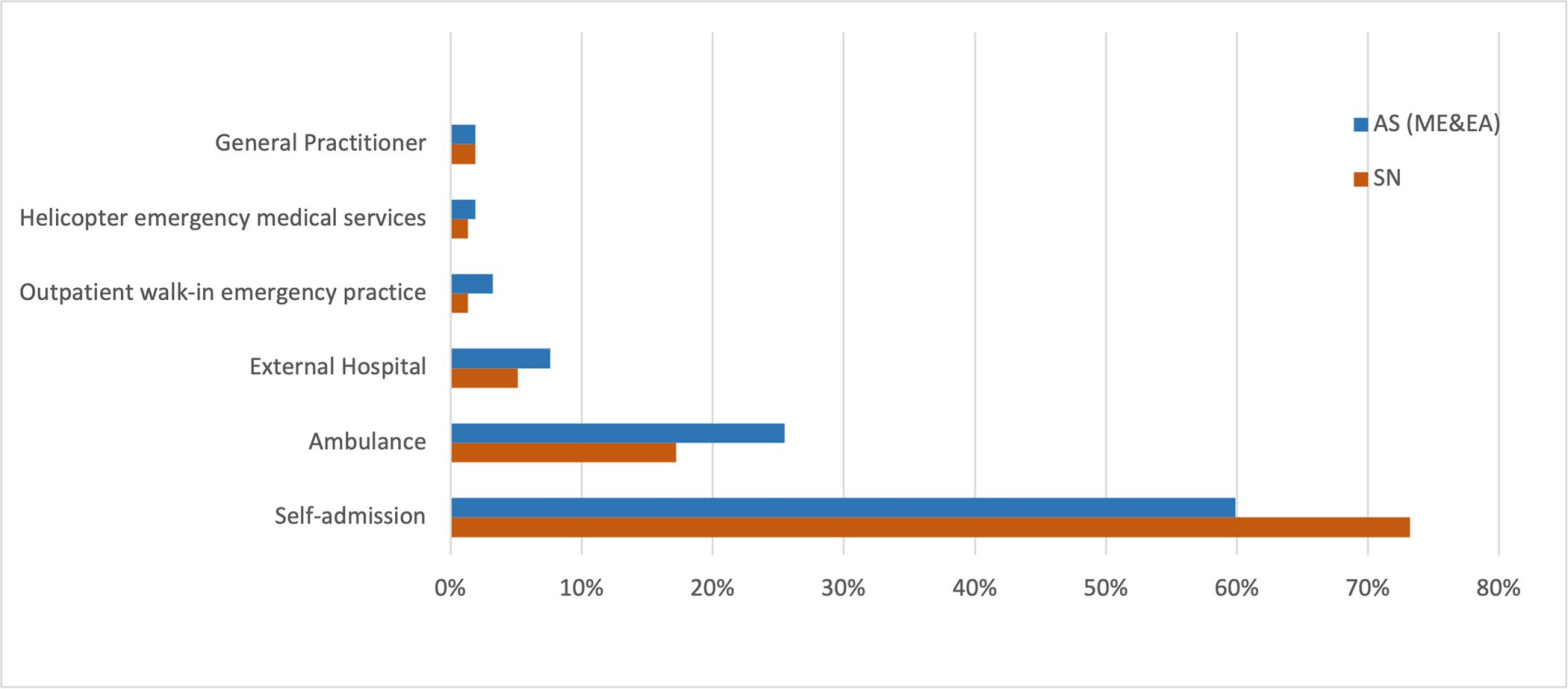
Type of admission to the emergency department (AS = Asylum Seeker patients; SN = Swiss national patients).

The distribution of the type of admission to the ED can be seen in Fig 1, with higher rates of self-admission in the Swiss patient group (73.2% vs. 59.9%) and higher rates of admission by ambulance within the AS patient group (25.5% vs. 17.2%). As regards the discharge status, the majority of patients in both groups were discharged home (SN = 82.8%; AS = 77.1%), fewer were hospitalized (SN = 15.3%; AS = 19.7%) and transfer to another hospital was rare (SN = 1.9%; AS = 2.5%).

**Fig 2:**
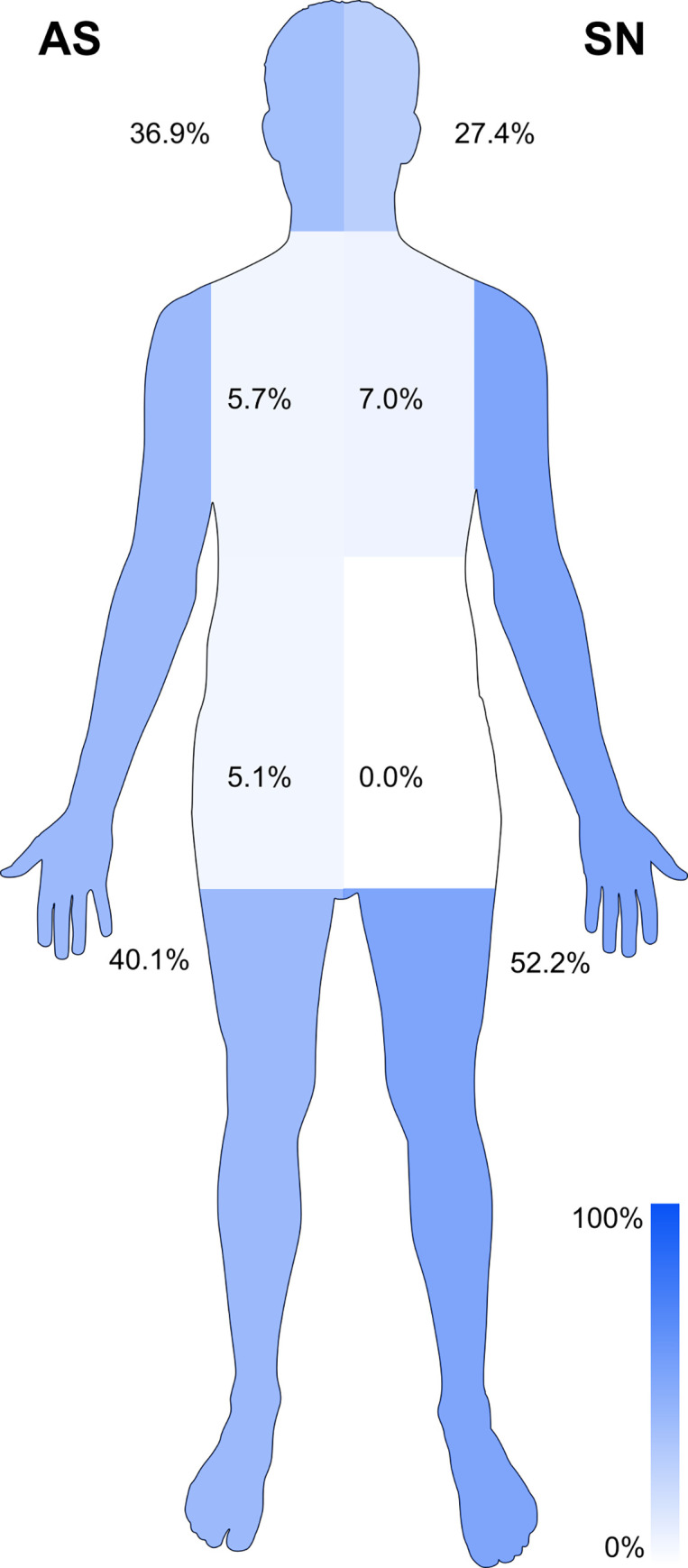
Comparison of the distribution of injured body regions in percent: AS = Asylum Seeker patients; SN = Swiss national patients, patients with injuries in multiple regions are not displayed (AS: 12.1% vs. SN: 13.4%).

The localization of injury differed significantly between the groups (χ 2-Test (4) = 13.0, p = 0.01 showed 2 cells with an expected frequencies of 4 (the remaining cells 80% were > 5), and also calculation of Fisher’s exact test = 13.6, p = 0.008). As illustrated in Fig 2, the AS patient group had more injuries in the area of the head/face/neck/throat (36.9% vs. 27.4%) and within the abdominal and pelvic area (5.1% vs. 0%), whereas the Swiss patient group had more injuries of the lower and upper extremities (52.2% vs. 40.1%).

The mechanism of injury differed significantly between the groups (χ 2-Test (7) = 30.9, p < 0.001 showed 2 cells with an expected frequency of 3 (the remaining cells 87.5% were > 5)). In addition, Fisher’s exact test gave a value of 31.3, p < 0.001. The main reasons for injuries within the Swiss patient group were accidents in everyday life or leisure, followed by falls, and injuries related to road traffic or work-related injuries, as can be seen in Table 3. Within the AS patient groups, the main reasons for injuries were assault, everyday life / leisure accidents, fall or sports related accidents.

**Table 3:** Comparison of mechanism, type of injury and use of substance (AS = Asylum Seeker group; SN = Swiss national group).

		AS		SN	
		Number	Percent	Number	Percent
**Mechanism of injury**	road traffic injury	13	8.3	23	14.6
	sports	23	14.6	23	14.6
	fall	21	13.4	28	17.8
	everyday / leisure	31	19.7	33	21.0
	work related	9	5.7	26	16.6
	assault	43	27.4	16	10.2
	attempted suicide	6	3.8	0	0.0
	other	11	7.0	8	5.1
**Type of injury**	fracture	15	9.9	17	11.7
	concussion	6	3.9	9	6.2
	cuts / bites / open wounds	37	24.3	56	38.6
	bruises / superficial	34	22.4	22	15.2
	sprain / strain / dislocation	29	19.1	20	13.8
	other	11	7.2	5	3.4
	multiple injuries	20	13.2	16	11.0
**Substance use**	alcohol	21	13.4	15	9.6
	drugs	1	0.6	3	1.9
	mixed	5	3.2	5	3.2
	none	130	82.8	134	85.4

The most frequent types of injuries in the AS patient group were: cuts/bites/open wounds, followed by bruises/superficial injuries, sprains/strains/dislocations and multiple injuries (see Table 3 for an overview). Similarly in the Swiss patient group, the most frequent injuries were cuts/bites/open wounds followed by bruises/superficial injuries, sprains/strains/dislocations and fractures. Yet these differences did not reach significance (χ 2-Test (6) = 11.4, p < 0.078).

The majority of the trauma patients in both groups did not use any substances before the injury occurred (> 84%). Within the users, the Swiss patients used slightly more drugs/medication or mixed substances with alcohol, whereas the AS used slightly more alcohol than the Swiss patients, yet the use of substances before the injury did not differ significantly between the groups (Fisher’s exact test = 1.97, p = 0.596).

## Discussion

The distribution of countries of origin and age groups of the AS study patients consulting the ED from EA and ME during the study period is similar to the distribution of AS living in Switzerland in 2018 [1]. Within our study population, we see a slightly higher proportion of AS men in comparison to the gender distribution of AS living in Switzerland (about 80% versus 65%). This is in line with the accident statistics of the Federal Office of Statistics in Switzerland, which also showed, that men more often had accidents in general and more frequently sought medical care for injuries in comparison to women [20]. In addition, previous studies of our ED demonstrated, that in AS patients consulting the ED, the proportion of men was around 65% and even higher in consultations for trauma (23.3% vs. 5.3%) [13, 15].

There were significant differences between the groups in the distribution of the severity of the injuries. The AS group had more minor and slightly more severe injuries, whereas the majority of the SN group had a moderate level of severity and fewer cases of minor and severe traumatic injuries. This is partly reflected in the type of admission to the ED, with higher rates of self-admission in the Swiss patient group and higher rates of admission by ambulance within the AS patient group. The higher rates of ambulance admission cannot only be explained by the difference in injury severity; other reasons might be lack of knowledge of the local health care system, difficulty accessing other primary care providers or different levels of perceived urgency [13]. In addition, the observed high rates of outpatient treatment for both groups underline the increasing role of EDs as primary care provider for the population served. This is in line with trends reported in the literature [21, 22].

The mechanisms of injury differed significantly between the two groups. In addition, in the AS group, higher rates were observed of intentional injuries, such as assault related injuries and attempted suicide. The term “assault” has to be interpreted with caution, as we did not distinguish between the type or cause of the assault, nor from whom it originated. In addition, the discrepancy in injury mechanism might be due to the difference in socio-economic status of the two groups, as regards having a job, driver’s license or access to motor vehicles, as the local Swiss population was more involved in road traffic and work related injuries. As few studies deal with trauma patterns of AS compared to the local population of their high income host countries, the comparability of our results is limited. According to an Ethiopian study, the most common cause of injury of the local population was found to be road traffic injuries (RTIs 51.4%) and therefore these were the leading causes for patients visiting the ED [19]. Another study from Turkey, which compared ED consultations of refugees to the local population, also showed similar results to ours, with a lower prevalence of RTIs among the refugee population [9]. In contrast to our study, they demonstrated higher prevalence of injuries from assault among the local population than in the refugee population. However, their classification was apparently based on acts of violence reported to the police and reports to the police were less likely to be made by refugees. Furthermore, there is growing evidence in the literature that suicidal ideation or rates of attempted suicide and self-harm are higher in asylum seekers after resettlement than in the local population [23–25]. Our study contributes to this crucial finding and the need for further research and specific monitoring of suicidal ideation and attempts to establish targeted and culturally appropriate public health interventions.

The localization of the injured body region differed significantly between the two groups, with more head injuries in the AS group. Similar results were shown in a study by Duzkoylu et al [9]. Although there was no difference regarding the use of substances between the two groups, it is noteworthy that almost one quarter of all patients was under the influence of alcohol or drugs. This worrisome finding might even be more pronounced in reality, since this item predominantly relies on self-reported data. High rates of injuries under substance abuse or alcohol require additional tailored primary prevention measures.

The type of injury did not differ between the groups, with cuts/bites/open wounds being the most frequent types of injuries, followed by bruises/superficial injuries and sprains/strains/dislocations in both groups. These findings are similar to those of Negussie et al., where the most common type of injury was also minor or superficial injury (e.g. bruises and minor cuts) [19]. Besides the care of cuts, some of which cannot be treated by primary care physicians (e.g. in the case of more severe cuts, which might require X-rays, extensive wound care or different types of bandages/splints), the other types of injury may very well be treated by primary care providers outside the emergency department. This underlines the increasing role of EDs as primary care provider. It requires either appropriate provision of human and material resources e.g. in form of incorporation into EDs of additional small GP-run walk in clinics, or outsourcing of this function to family doctors in the region. In addition, it demonstrates the need for an educational campaign on how the health care system works and specifically on the scope of medical treatment available by primary care physicians.

There are also several limitations applying to the study performed. As the study had a retrospective design at a single center university ED with a relatively small sample size, this reduces the generalizability of the results to other emergency departments or hospitals. The generalizability is additionally reduced in that only patients originating from ME or EA were included in the study population. By including the two largest asylum-seeking populations, we attempted to create a sample similar to the population structure. Furthermore, there is a risk for selection bias by choosing the next Swiss Trauma patient for comparison. However, we chose this method in order to counteract a temporal bias e.g., consultation during night and weekends vs. daytime or winter vs. summer, as this can severely influence the type and pattern of injury. Another limitation might be that we cannot make any statement on the AS-patients length of stay in Switzerland by the time of their medical consultation, since the length of stay is not routinely documented in the medical record. In addition, further research is needed on the underlying causes for the identified differences between the two patient groups in injury patterns and characteristics, as well as subpopulation analysis, as these were not the subject of investigation in our study. Despite these limitations, we believe that insights about injury patterns and characteristics of asylum seeking compared to the local population can help stakeholders to adjust and prepare trauma care settings and to adapt injury prevention campaigns.

## Conclusions

There are differences in injury patterns and characteristics of physical trauma patients between AS and the local population of the high income hosting country. Disparities in the type of admission, level of severity, localization and mechanism of injuries suggest it may be necessary to adapt injury prevention strategies to the respective target population. The overall high usage of substances by injured patients requires further public health attention. In addition, the observed high rates of outpatient treatment for both groups underline the increasing role of EDs as primary care provider for the population served.

## Abbreviations

AS: Asylum Seeker
CH: Switzerland
ED: Emergency Department
EA: Eastern Africa
ME: Middle East
SN: Swiss national
LOS: Length of stay
STS: Swiss Triage Scale
ICD-10: International Statistical Classification of Diseases and Related Health Problems Version 10
IQR: Interquartile range
RTI: Road traffic injurie
GP: General practitioner
